# Causal effects of endometriosis on SLE, RA and SS risk: evidence from meta-analysis and Mendelian randomization

**DOI:** 10.1186/s12884-024-06347-9

**Published:** 2024-02-23

**Authors:** Tianyou Tang, Yi Zhong, Sipei Xu, Huilin Yu

**Affiliations:** 1 Children’s Hospital of Chongqing Medical University, Chongqing, China; 2 The First Medicine College, Chongqing Medical University, Chongqing, China; 3 The Second Medicine College, Chongqing Medical University, Chongqing, China

**Keywords:** Endometriosis, Systemic lupus erythematosus (SLE), Rheumatoid arthritis (RA), Sjögren’s syndrome (SS), Meta-analysis, Mendelian randomization

## Abstract

**Background:**

Endometriosis is an underdiagnosed disorder that affects an estimated 6-10% of women of reproductive age. Endometriosis has been reported in epidemiological studies to be associated with autoimmune diseases. However, the relationship remains controversial.

**Methods:**

A meta-analysis of observational studies was undertaken to evaluate the risk of autoimmune diseases in patients with endometriosis. The relevant studies were retrieved via the databases Medline, Embase and Web of Science until July 20, 2023. Mendelian randomization (MR) was subsequently utilized to scrutinize the causal influence of genetic predisposition toward endometriosis on three autoimmune diseases.

**Results:**

The meta-analysis findings revealed a relationship between endometriosis and the onset of SLE (cohort studies: RR = 1.77, 95% confidence interval (CI): 1.47–2.13, I^2^ = 0%; Case-control and cross-sectional studies: OR = 5.23, 95% CI: 0.74–36.98, I^2^ = 98%), RA (cohort studies: RR = 2.18, 95% CI: 1.85–2.55, I^2^ = 92%; Case-control and cross-sectional studies: OR = 1.40, 95% CI: 1.19–1.64, I^2^ = 0%) and SS (cohort studies: RR = 1.49, 95% CI: 1.34–1.66, I^2^ = 0%). Similarly, in our MR study, the results of the inverse-variance-weighted (IVW) model suggested that genetic predisposition to endometriosis was causally associated with an increased risk for SLE (OR = 1.915, 95% CI: 1.204–3.045, *p* = 0.006) and RA (OR = 1.005, 95% CI: 1.001–1.009, *p* = 0.014).

**Conclusions:**

Both our meta-analysis and MR study indicate that endometriosis increases the risk of autoimmune diseases. These findings not only broaden our understanding of the genetic mechanisms underlying the comorbidity of endometriosis and autoimmune diseases, but also offer a new strategy for autoimmune disease prevention.

**Supplementary Information:**

The online version contains supplementary material available at 10.1186/s12884-024-06347-9.

## Introduction

Endometriosis is an estrogen-dependent chronic inflammatory disease that affects approximately 7–10% of women worldwide. It is characterized by two main symptoms: pelvic pain and infertility [1]. Serval studies have indicated that endometriosis is associated with numerous diseases, including gastrointestinal diseases, malignancies, cardiovascular diseases, mental disorders and autoimmune diseases [2, 3]. The pathogenesis of endometriosis remains unclear, but the retrograde menstruation theory is currently widely accepted [4].

Autoimmune diseases affect 3–5% of the population, with some being organ-specific, like RA, and others involving multiple organs, such as SLE [5]. There have been many review articles discussing the immunological aspects of endometriosis [6, 7]. They believe that the changes in cell-mediated and humoral immunity in patients with endometriosis may be the reason for the increased risk of autoimmune diseases. However, there are few articles based on population-based observational studies. In an attempt to understand more about the risk of autoimmune diseases in endometriosis, we embarked on a meta-analysis by including cohort studies, cross-sectional studies and case–control studies.

Mendelian randomization (MR) analysis employs genetic variation as an instrumental variable, enabling the evaluation of relationships between an exposure and an outcome. By leveraging the random distribution of genetic variation, MR helps eliminate confounding factors and reverse causation, thus simulating the randomization process seen in a randomized controlled experiment [8–10]. The degree of the connection and the direction of causality between endometriosis and autoimmune diseases were evaluated in this study using MR. In this study, we employed a meta-analysis in conjunction with MR analysis to elucidate the causal relationship, strength of association, and direction of causality between endometriosis and three autoimmune diseases.

## Methods

### Meta-analysis

#### General information

We performed this meta-analysis following the Preferred Reporting Items for Systematic Reviews and Meta-analyses (PRISMA) guidelines [11]. The study protocol was registered at PROSPERO (CRD42023444650). (https://www.crd.york.ac.uk/PROSPERO/).

#### Search strategy

A search strategy was developed as presented in (Datasheet1: Table S1). Two researchers (TYT and YZ) conducted a comprehensive electronic literature search of the PubMed, Web of Science, and Embase databases from their inception until July 2023. No restrictions were applied regarding geographic area, language, or publication status. Additionally, the researchers manually reviewed the reference lists of relevant articles to identify any additional studies that may have been missed in the initial search.

#### Study selection

Two of the authors (TYT and YZ) initially screened the titles and abstracts of the studies to exclude those that appeared irrelevant. Then, they thoroughly read the full texts of the remaining studies to further exclude any studies that did not meet the eligibility criteria. Any disagreements between the two authors were resolved through discussion.

Eligible articles for this study had to satisfy the following criteria: 1) having a cohort, case–control or cross-sectional study design and published in English, 2) comparing the risk of autoimmune disease among women with/without endometriosis, 3) providing data on odds ratio (OR), risk ratio (RR), hazard ratio (HR), standardized incidence ratio (SIR), incidence rate ratio (IRR) for autoimmune disease. (Table 1).

**Table 1:** PICOS criteria for inclusion of studies

Participants	The general population
Intervention/exposure	Endometriosis
Comparison	People without endometriosis
Outcome	Autoimmune diseases risk
Study design	Cohort, case–control and cross-sectional study

#### Data extraction

Two authors (TYT and YZ) independently extracted data and a consensus was reached in case of any inconsistency.

Using a pre-designed data extraction form, the following information was meticulously recorded: title, the name of the primary author, publication year, country, average age, duration of follow-up, sample size, outcome assessment, risk estimate, corresponding 95% confidence intervals.

Assessing the Risk of Bias.

The Newcastle–Ottawa quality assessment scale (NOS) was used to evaluate the methodological quality of cohort study and case–control study included in the analysis [12]. In the absence of established standard criteria, we categorized studies with 0–3 stars, 4–6 stars, or 7–9 stars as low-quality, moderate-quality, or high-quality, respectively. To evaluate the methodological quality of cross-sectional studies, we used the criteria provided by the Agency for Healthcare Research and Quality (AHRQ) [13]. Each item in the assessment was assigned a score of '0' if it was answered as 'NO' or 'UNCLEAR', and a score of '1' if it was answered as 'YES'. The total score for each study was then calculated. Based on the total score, the article quality was categorized as low-quality (0–3), moderate-quality (4–7), or high-quality (8–11). Disagreements were resolved through discussion.

#### Statistical analysis

The meta-analysis was conducted using Review Manager 5.4. For cross-sectional and case–control studies, raw data were extracted to compute a odds ratio (OR) accompanied by 95% confidence intervals (CIs). For cohort studies, SIR, IRR and HR were treated as the relative risk (RR), and the pooled RR with a 95% confidence interval was calculated [14, 15]. To ensure a more accurate assessment of the relationships between endometriosis and SLE, RA and SS, categorical meta-analyses were conducted. The I2 statistic was used to evaluate the degree of heterogeneity among the included studies. If the I2 value exceeded 50% or the p-value was less than 0.05, indicating a high level of heterogeneity, a random-effects model was employed. Conversely, if the I2 value was below 50% or the p-value was greater than or equal to 0.05, a fixed-effect model was used in the meta-analysis. This approach helps to account for heterogeneity and provide more reliable results.

### Mendelian randomization

#### Study design

**Fig. 1:**
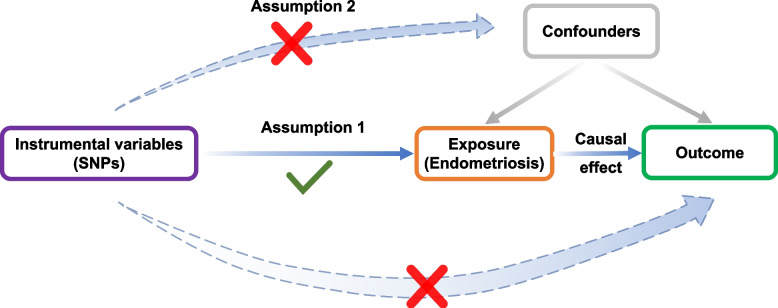
Diagram for key assumptions of MR analyses. Exposure SNPs were used as the genetic instruments to investigate the causal effect of endometriosis on outcome. The directional arrows indicates that the genetic instruments (SNPs) are associated with the exposure and can only influence the outcome through the exposure. Dashed lines represent that the genetic instruments (SNPS) are independent of any confounding variables affecting the results

The Mendelian randomization (MR) method is based on three key assumptions, which are summarized in Fig. 1. First, the selected SNPs must be significantly correlated with the exposure factor. Second, SNPs must be independent of potential confounding factors. Third, SNPs should not have a direct association with outcome.

#### Data source

For the study of endometriosis, we retrieved genome-wide association study (GWAS) summary data from Nilufer R et al. (PMID: 36,914,876) [16]. In this GWAS, a total of 60,694 patients with endometriosis and 701,926 controls of European and East Asian descent were included by the authors. Summary statistics of SLE are from Wang YF et al. (PMID: 33,536,424), including up to 12,653 participants (4,222 cases and 8,431 controls) of East Asian ancestry [17]. Summary statistics of RA are from Neale Lab, including up to 337,159 participants (3,730 cases and 333,429 controls) of European ancestry. The summary statistics of SS were obtained from the FinnGen consortium release data (1,290 cases and 213,145 controls).

#### Statistical analysis

Cochran's Q test was used in this MR study to determine whether there was variability in estimates of specific genetic variants [18]. Inverse variance weighted (IVW) analysis was the main technique employed [19]. In addition to IVW, further analyses were carried out utilizing the weighted median method [20], simple mode, weighted mode, and MR-egger regression method [21]. Finally, to guarantee the accuracy of the results, we tested and calibrated horizontal pleiotropic outliers in the IVW model using MR pleiotropy residual sum and outlier (MR-PRESSO) [22].

#### Sensitivity analysis

To detect potential pleiotropy, we conducted the MR-Egger test and interpreted a P-value greater than 0.05 for the MR-Egger intercept as an absence of horizontal pleiotropy [23]. To assess the stability of the results, leave-one-out sensitivity analyses were conducted, wherein a single SNP was excluded in each iteration. This analysis helps determine if any single SNP is driving the observed associations. Funnel plots and forest plots were generated to visually explore the existence of pleiotropy, which is when a genetic variant affects multiple traits or outcomes. A two-sided p-value of less than 0.05 was considered as suggestive of significance. All the analyses were performed using the "Two-Sample-MR" and "MR-PRESSO" packages in R software, specifically Version 4.2.3.

## Results

### Meta-analysis

#### Study selection and characteristics

**Fig. 2:**
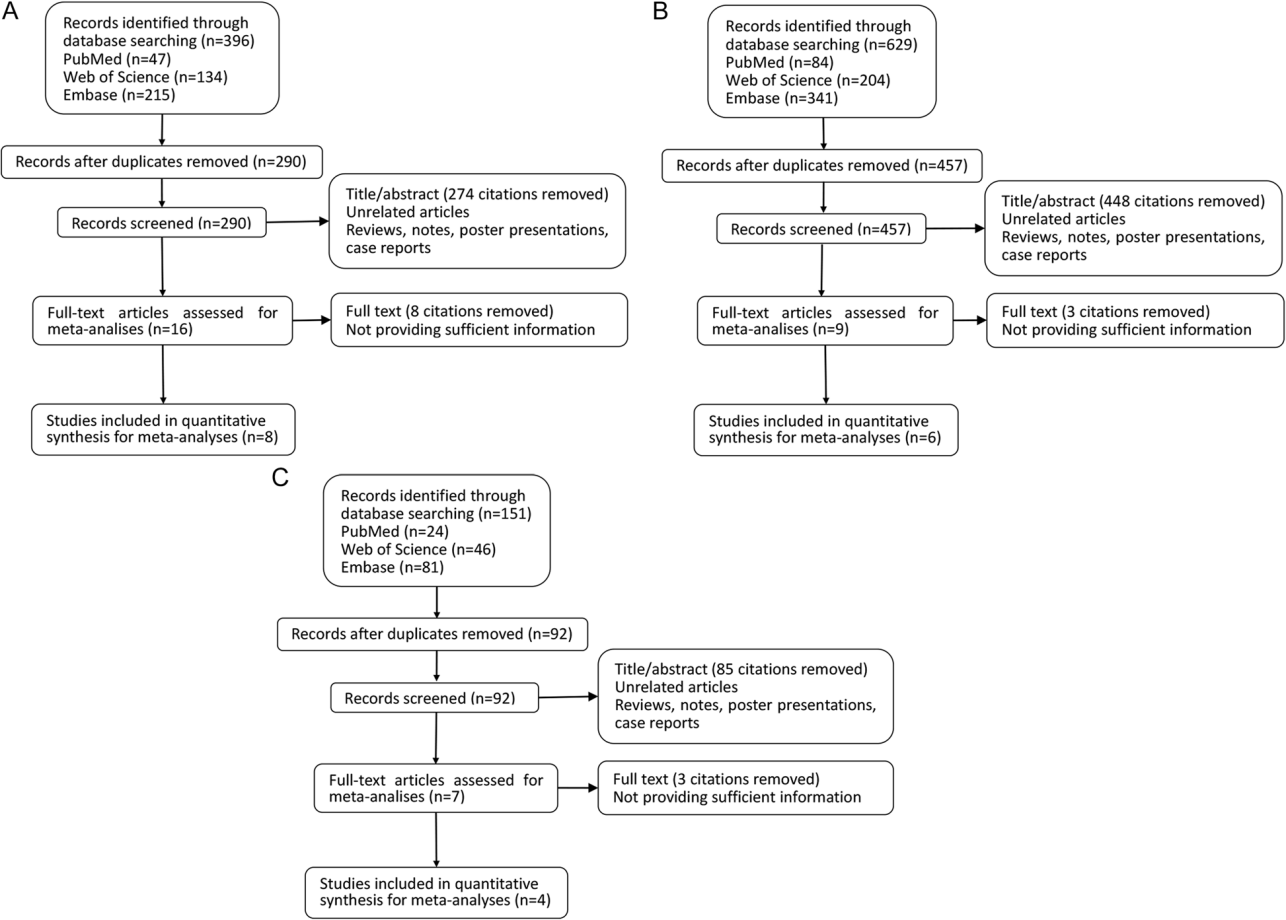
The flowchart of meta-analysis

A flowchart of the process of choosing the specific literature is shown in Fig. 2. Tables 2 and 3 provide a detailed summary of key features for the 13 included research. In brief, 3 (23.1%) investigations were conducted in North America, 4 (30.8%) in Europe, and 6 (46.2%) in Asia. In terms of study design, one (7.8%) study was cross-sectional, four (30.8%) were case–control studies, and eight (61.5%) were cohort studies (four prospective cohort studies and four retrospective cohort studies).

**Table 2:** Characteristics of case–control and cross-sectional studies

Citation	Country	Study period	Study design	Effect estimates	Study population (*n*)	Diagnosis criteria	
						Endometriosis	Autoimmune disease
Matorras et al. (2007) [27]	Spain	1990- 2004	Case–control study	SLE: OR = 2.9 (95%CI:0.27–32.57) *P* > 0.05 SS: OR、95%CI:not calculable *P* > 0.05	Case: 342 Control: 501	Histology	Clinical interview and medical records according to the ACR criteria
Yoshii et al. (2021) [31]	Japan	2011- 2018	Case–control study	SLE: IRR = 1.35 (95%CI:0.99–1.84) RA: IRR = 1.31 (95%CI:1.05–1.64)	Case: 30,516 Control: 120,976	ICD-10-CM	ICD-10-CM
Porpora et al. (2019)	Italy	2014- 2017	Retrospective case–control study	SLE: OR = 8.63 (95%CI:1.07–69.91) *P* = 0.01	Case: 148 Control: 150	Laparoscopy and histology	Antinuclear antibodies, extractable nuclear antigen, anti-cardiolipin antibodies, antiphospholipid antibodies, and lupus anticoagulant
Sinaii et al. (2002) [30]	USA, Canada	1988	Cross-sectional study	SLE: OR = 20.7 (95%CI:14.3–29.9) *P* < 0.0001 RA: OR = 1.5 (95%CI:1.2–1.9) *P* = 0.001 SS: OR = 23.9 (95%CI:15.5–36.5) *P* < 0.0001	Study sample: 3680	Self-reports of laparoscopy/laparotomy	Self-reported physician diagnosis

**Table 3:** Characteristics of cohort studies

Citation	Country	Study period	Study design	Effect estimates	Study population (*n*)	Diagnosis criteria	
						Endometriosis	Autoimmune disease
Nielsen et al. (2011) [28]	Denmark	1977- 2007	Retrospective cohort study; 12.1 years follow-up	SLE: SIR:1.6 (95%CI:1.2–2.1) SS: SIR:1.6 (95%CI:1.3–2.0)	Exposure: 37,661	ICD8:codes 62,530–62539 ICD10: code group N80	Medical records according to the ACR criteria
Harris et al. (2016a) [25]	USA	1989- 2011	Prospective cohort study; 22 years follow-up	SLE: HR = 1.61 (95%CI:0.88–2.92) *P* < 0.05 RA: HR = 1.16 (95%CI:0.84–1.59) *P* < 0.05	Exposure: 6434 Control: 108,019	Self-reported laparoscopy	Medical records according to the ACR criteria
Lin et al. (2020) [26]	Taiwan	2000- 2012	Retrospective cohort study; patients were followed until diagnosed with SLE, death(means = 8.1 years)	SLE: HR = 1.86 (95%CI:1.36–2.53) *P* < 0.0001	Exposure: 17,779 Control: 17,779	ICD-9-CM	Medical records according to board-certified rheumatologists
Fan et al. (2021) [24]	Taiwan	2000- 2011	Retrospective cohort study; 12 years follow-upar	SLE: HR = 2.37 (95%CI:1.35–4.14)	Exposure: 16,758 Control: 16,758	ICD-9-CM	ICD-9-CM
Merlino et al. (2003) [33]	USA	1986- 1997	Prospective cohort study; 11 years follow-up	RA: RR = 1.59 (95%CI:0.82–3.08)	/	Self-reported physician diagnosis	Self-reported physician diagnosis
Chen et al. (2020)	Taiwan	2000- 2012	Prospective cohort study; patients was followed until the appearance of RA, their removal from the NHIP, death, or the end of 2013 (means = 8.1 years)	RA: HR = 3.71 (95%CI: 2.91–5.73) P = 0.77	Exposure: 17,913 Control: 17,913	ICD-9-CM	ICD-9-CM
Xue et al. (2020)	Taiwan	2000- 2013	Prospective cohort study; 13 years follow-up	RA: HR = 1.75 (95%CI: 1.27–2.41) *P *< 0.05	Exposure: 14,463 Control: 14,463	ICD-9-CM	ICD-9-CM
Chao et al. (2022) [35]	Taiwan	2000- 2012	Retrospective cohort study	SS: HR = 1.45 (95%CI:1.27–1.65) *P* < 0.001	Exposure: 14,733 Control: 58,932	ICD-9-CM	ICD-9-CM

#### Quality assessment

According to the information provided, the researchers used the Agency for Healthcare Research and Quality (AHRQ) rating criteria to assess the quality of cross-sectional studies included in their analysis. Additionally, the Newcastle–Ottawa Scale (NOS) was used to evaluate the quality of case–control and cohort studies. The results of this assessment can be found in Tables 4 and 5, which presumably shows that all the publications included in the study were rated as high or moderate quality based on the use of AHRQ and NOS criteria.

**Table 4:** NOS assessment for case–control and cross-sectional studies

NEWCASTLE—OTTAWA QUALITY ASSESSMENT SCALE					
Author	Selection	Comparability	Exposure	Total score	Quality grade
Nielsen et al. (2011) [28]	2	1	3	6	moderate
Harris et al. (2016a) [25]	1	2	3	7	high
Lin et al. (2020) [26]	4	1	2	7	high
Fan et al. (2021) [24]	4	2	3	9	high
Merlino et al. (2003) [33]	4	2	3	9	high
Chen et al. (2020)	4	1	2	7	high
Xue et al. (2020)	4	2	3	9	high
Chao et al. (2022) [35]	3	2	2	7	high
Matorras et al. (2007) [27]	3	1	3	7	high
Porpora et al. (2019)	2	1	3	6	moderate
Yoshii et al. (2021)	2	2	2	6	moderate

**Table 5:** AHRQ assessment for cross-sectional studies

Agency for Healthcare Research and Quality		
Author	Total score	Quality grade
Sinaii et al. (2002) [30]	6	moderate
Shafrir et al. (2021)	8	high

#### Data synthesis

##### Endometriosis and SLE

**Fig. 3:**
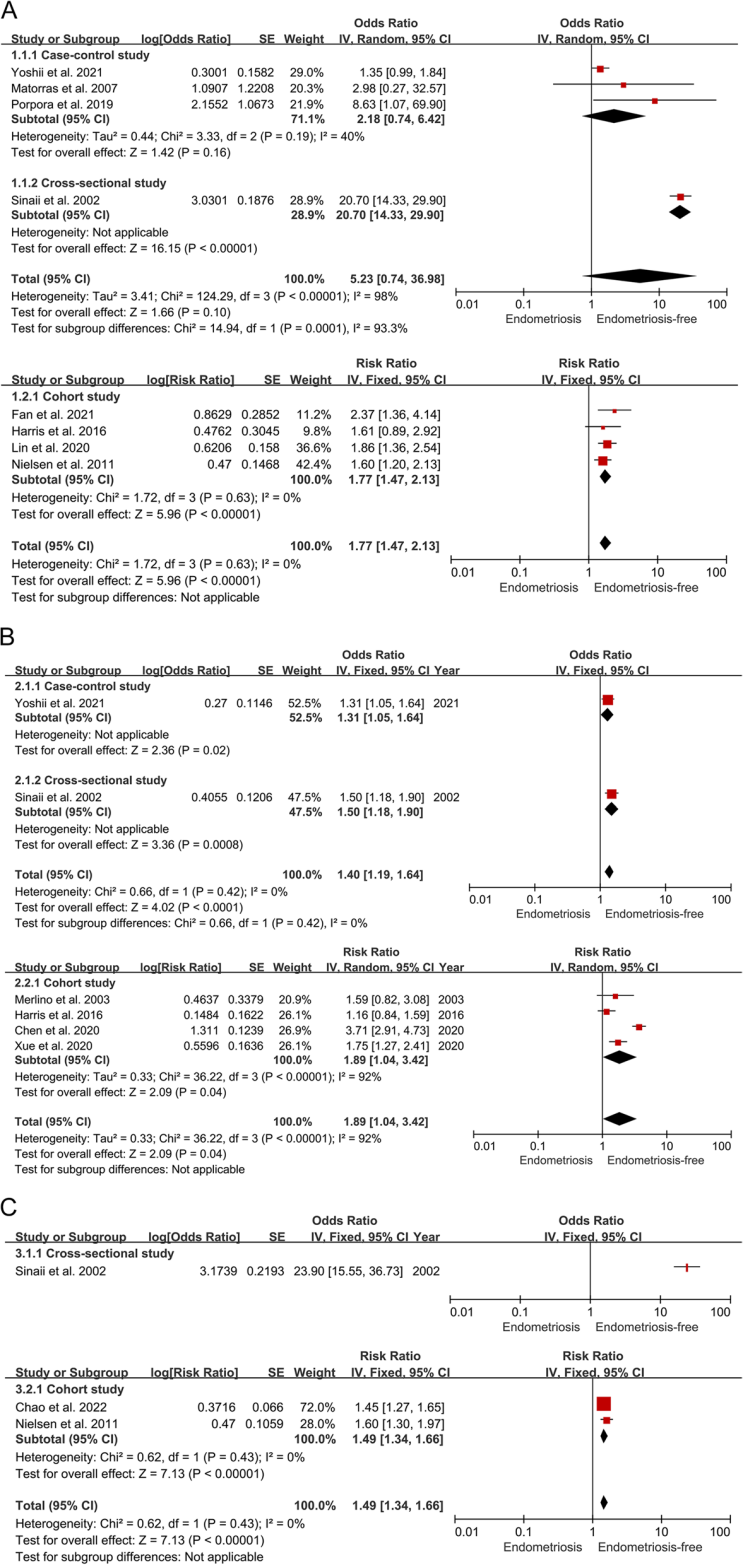
Forest plots of studies. (**A**) the association of endometriosis with SLE risk; (**B**) the association of endometriosis with RA risk; (**C**) the association of endometriosis with SS risk

Eight studies were included in a meta-analysis comparing the risk of SLE in individuals with endometriosis and those without endometriosis, involving 477,501 individuals and 113,318 endometriosis cases [24–31]. For case–control and cross-sectional studies, we did not find a significant association between the two conditions. For cohort studies, with low heterogeneity, the pooled risk of SLE was greater in individuals with endometriosis than in those without endometriosis (RR = 1.77, 95% CI 1.47–2.13; I2 = 0%) (Fig. 3).

##### Endometriosis and RA

Six studies were included in a meta-analysis comparing the risk of RA in individuals with endometriosis and those without endometriosis, involving 434,377 individuals and 73,006 endometriosis cases [25, 30–34]. Case–control and cross-sectional studies have described the association between endometriosis and RA (OR = 1.40, 95% CI 1.19–1.64; I2 = 0%). For cohort studies, with high heterogeneity, we observed similar trends in the results (RR = 1.89, 95% CI 1.04–3.42; I2 = 92%) (Fig. 3).

##### Endometriosis and SS

Three studies were included in a meta-analysis comparing the risk of SS in individuals with endometriosis and those without endometriosis, involving 215,006 individuals and 56,074 endometriosis cases [28, 30, 35]. For cohort studies, with low heterogeneity, the pooled risk of SS was greater in individuals with endometriosis than in those without endometriosis (RR = 1.49, 95% CI 1.34–1.66; I2 = 0%) (Fig. 3).

### Mendelian randomization study

#### Genetic associations between endometriosis and SLE, RA, and SS risk

**Fig. 4:**
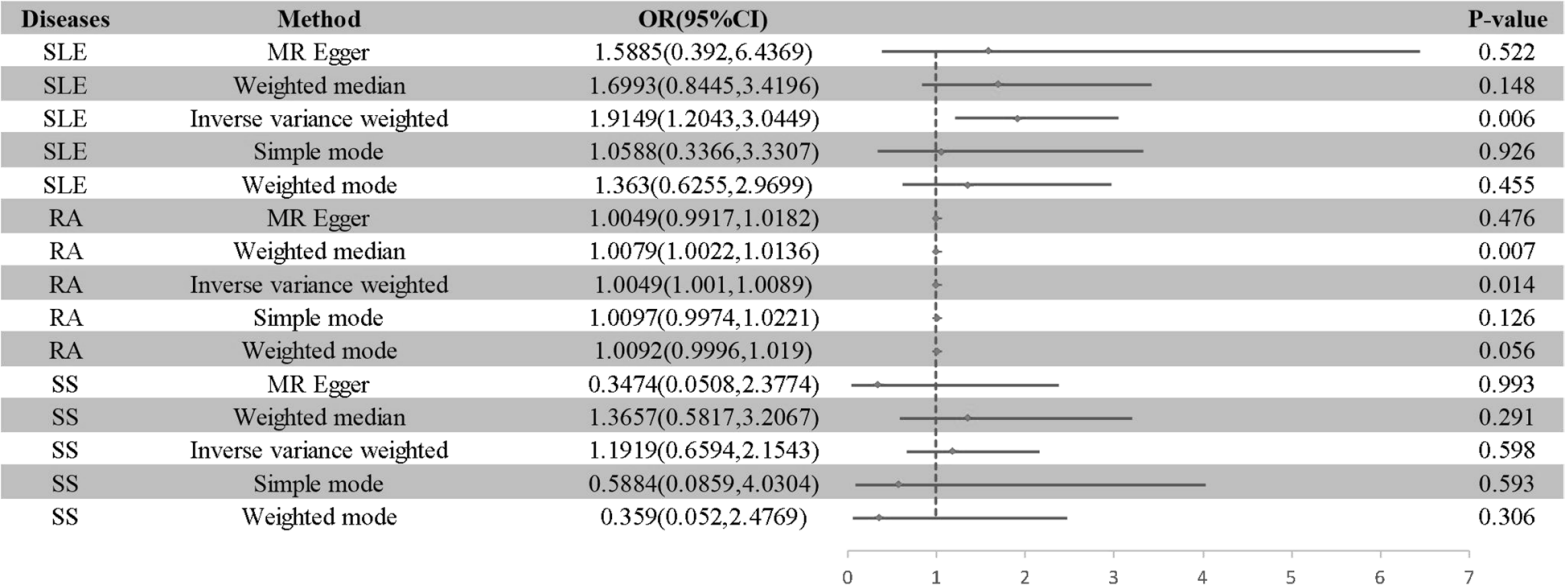
Associations of genetic predisposition to endometriosis with SLE, RA, and SS risk

Using the random-model IVW, we discovered a link between each standard rise in endometriosis risk and a faster development to SLE (OR = 1.915, 95% CI: 1.204–3.045, *p* = 0.006) and RA (OR = 1.005, 95% CI: 1.001–1.009, *p* = 0.014). However, no causal relationship was found between endometriosis and SS. (Fig. 4).

#### Sensitivity analysis

Cochran's Q test findings revealed that these SNPs exhibited no heterogeneity. We found no evidence of directional pleiotropy using MR Egger intercepts and MR-PRESSO (Datasheet 2). The leave-one-out analysis produced a stable result when each SNP was eliminated, as shown in Datasheet 3.

## Discussion

Many previous studies have found a link between endometriosis and autoimmune disorders, a literature review was compiled on the topic (Datasheet1:Table S2). We employ meta-analysis and MR to investigate causative associations between endometriosis and SLE, RA, and SS risk. To our knowledge, the latest and probably only meta-analysis was published in 2019 [36]. Due to the inability of cross-sectional and case–control studies to resolve the issue of the temporal relationship between endometriosis and autoimmune diseases, their research faced challenges in determining the sequence of disease development and manifestation, as well as potential causal relationships. Compared to this study, our study comprises more studies in patients with endometriosis, including newer and larger cohort studies. We aimed to investigate the causal effect of endometriosis on SLE, RA and SS. As the first report to employ MR in investigating the causal association between endometriosis and the risk of SLE, RA, and SS, our study eliminates confounding factors and reverse causality effects, which may yield more reliable results.

The meta-analysis findings reveal that endometriosis patients are more likely to develop SLE and SS, which is consistent with the results of the meta-analysis from 2019. However, in contrast to previous studies, we found that endometriosis also increases the risk of RA. The discrepancy in findings between the two studies may be due to the fact that the 2019 study only had two cohort studies available for inclusion. We observed that only a limited number of studies accounted for the effects of confounding factors, which may influence the level of the risk. Smoking, alcohol usage, caffeine consumption, and a lack of exercise have all been linked to an increased risk of endometriosis [37]. These lifestyle and environmental factors have also been linked to an increased susceptibility to cancer occurrence [38]. At the same time, the number of included studies was relatively small, which poses a risk of generating spurious associations. Therefore, we encourage more researchers to further investigate the association by employing prospective cohort study designs.

We also utilize a two-sample MR approach to further investigate the impact of endometriosis on the risk of autoimmune diseases. We employ independent loci associated with endometriosis identified from the largest available genome-wide association studies (GWAS) to date. All SNPs were identified in the PhenoScanner database (http://www.phenoscanner.medschl.cam.ac.uk/) to exclude SNPs related to confounding factors. With MR, we found that endometriosis is linked with a higher risk of SLE and RA, which supports prior meta-analyses.

The imbalance of the immune system may explain the the observed impact of endometriosis on autoimmune diseases in clinical practice [39–41]. Previous studies have demonstrated that elevated expression of IL-6, IL-15, and TGF-β1 in patients with endometriosis can reduce the activity of NK cells [42–44]. It has been observed that patients with endometriosis often have an increase in neutrophils and macrophages in their peritoneal fluid [45, 46]. In the latest meta-analysis to date, Riccio et al. suggested that there is an increase in B lymphocytes and excessive production of autoantibodies in endometriosis [47]. These alterations play an important role in mediating the pathogenesis of autoimmune diseases [48–50].

Estrogen also plays an important role in the development of endometriosis and autoimmune diseases. Endometriosis is an estrogen-dependent disease, and the disruption of estrogen signaling leads to hormonal imbalance, which causes its symptoms [51]. Targeting estrogen is still considered the optimal approach for controlling the progression and inflammation of endometriosis [52]. Estrogen has also been found to regulate the immune system and contribute to the transduction pathways of autoimmunity by activating its nuclear receptor AhR [53]. Estrogen raises the risk of autoimmune diseases by raising the generation of type 1 interferon and promoting the survival of B cells that create pathogenic IgG autoantibodies [54].

Firstly, pleiotropy has always been an important issue in Mendelian randomization. However, neither the MR-Egger nor the MR-PRESSO analyses revealed any indication of horizontal pleiotropy, indicating a very low level of pleiotropic bias. Secondly, the observational studies collected in our meta-analysis did not consider the influence of mediation effects. For example, patients with endometriosis exhibited an increased susceptibility to sedentary behavior [3]. Sedentary behavior is also acknowledged as a risk factor for autoimmune diseases [55]. Finally, our study did not examine the effect of endometriosis on the prognosis of autoimmune disorders due to a lack of data. Therefore, more studies are warranted to elucidate the possible relation between the two conditions. 

### Supplementary Information

**Supplementary Material 1.**

**Supplementary Material 2.**

**Supplementary Material 3.**

## Data Availability

The 42 SNPs selected for endometriosis are provided in Datasheet2 Table S1. The data that support the findings of this study are openly available in an open website (https://gwas.mrcieu.ac.uk/). All data generated or analysed during this study are included in this published article and datasheet.

## Abbreviations

Fig: Figure
GWAS: Genome-wide association study
IVW: Inverse-variance-weighted
MR: Mendelian randomization
