# Supplementary material for: Causal effects of endometriosis on SLE, RA and SS risk: evidence from meta-analysis and Mendelian randomization
Source: BMC Pregnancy Childbirth. 2024 Feb 23;24:162. doi: 10.1186/s12884-024-06347-9 (PMC10885476; doi:10.1186/s12884-024-06347-9)
Supplement: Supplementary file 1 — Supplementary Material 1. [file 12884_2024_6347_MOESM1_ESM.docx]

Table S1

Lupus Erythematosus, Systemic

Database: PubMed

Search date: July 20, 2023

Results: 47 articles

Search strategy:

((((Endometriosis) OR (Endometrioses)) OR (Endometrioma)) OR (Endometriomas)) AND ((((((Lupus Erythematosus, Systemic) OR (Systemic Lupus Erythematosus)) OR (Lupus Erythematosus Disseminatus)) OR (Libman-Sacks Disease)) OR (Disease, Libman-Sacks)) OR (Libman Sacks Disease))

Database: Web of Science

Search date: July 20, 2023

Results: 134 articles

Search strategy:

1: TS=(Endometriosis) OR TS=(Endometrioses) OR TS=(Endometrioma) OR TS=(Endometriomas)

2: TS=(Lupus Erythematosus, Systemic) OR TS=(Systemic Lupus Erythematosus) OR TS=(Lupus Erythematosus Disseminatus) OR TS=(lipman-Sacks Disease) OR TS=(Disease, lipman-Sacks) OR TS=(lipman Sacks Disease)

3: #1 AND #2

Database: Embase

Search date: July 20, 2023

Results: 215 articles

Search strategy:

#1 'endometriosis'/exp OR endometriosis OR endometrioses OR endometrioma OR endometriomas

#2 'lupus erythematosus, systemic'/exp OR 'lupus erythematosus, systemic' OR (lupus AND erythematosus, AND systemic) OR (systemic AND lupus AND erythematosus) OR (lupus AND erythematosus AND disseminatus) OR ('libman sacks' AND disease) OR (disease, AND 'libman sacks') OR (libman AND sacks AND disease)

#3 #1 AND #2

Arthritis, Rheumatoid

Database: PubMed

Search date: July 20, 2023

Results: 84 articles

Search strategy:

((((Endometriosis) OR (Endometrioses)) OR (Endometrioma)) OR (Endometriomas)) AND ((Arthritis, Rheumatoid) OR (Rheumatoid Arthritis))

Database: Web of Science

Search date: July 20, 2023

Results: 204 articles

Search strategy:

1: TS=(Endometriosis) OR TS=(Endometrioses) OR TS=(Endometrioma) OR TS=(Endometriomas)

2: TS=(Arthritis, Rheumatoid) OR TS=(Rheumatoid Arthritis)

3: #1 AND #2

Database: Embase

Search date: July 20, 2023

Results: 341 articles

Search strategy:

#1 'endometriosis'/exp OR endometriosis OR endometrioses OR endometrioma OR endometriomas

#2 'arthritis, rheumatoid'/exp OR 'arthritis, rheumatoid' OR (('arthritis,'/exp OR arthritis,) AND rheumatoid) OR (rheumatoid AND arthritis)

#3 #1 AND #2

Sjogren's Syndrome or Sjogrens Syndrome

Database: PubMed

Search date: July 20, 2023

Results: 24 articles

Search strategy:

((((Endometriosis) OR (Endometrioses)) OR (Endometrioma)) OR (Endometriomas)) AND ((((((Sjogren's Syndrome) OR (Sjogrens Syndrome)) OR (Syndrome, Sjogren's)) OR (Sjogren Syndrome)) OR (Sicca Syndrome)) OR (Syndrome, Sicca))

Database: Web of Science

Search date: July 20, 2023

Results: 46 articles

Search strategy:

1: TS=(Endometriosis) OR TS=(Endometrioses) OR TS=(Endometrioma) OR TS=(Endometriomas)

2: TS=(Sjogren's Syndrome) OR TS=(Sjogrens Syndrome) OR TS=(Syndrome, Sjogren's) OR TS=(Sjogren Syndrome) OR TS=(Sicca Syndrome) OR TS=(Syndrome, Sicca)

3: #1 AND #2

Database: Embase

Search date: July 20, 2023

Results: 81 articles

Search strategy:

#1 'sjogren syndrome'/exp OR 'sjogren syndrome' OR (sjogren AND ('syndrome'/exp OR syndrome)) OR (syndrome, AND sjogren) OR (sicca AND syndrome) OR (syndrome, AND sicca) OR (sjogrens AND syndrome)

#2 'sjogren syndrome'/exp OR 'sjogren syndrome' OR (sjogren AND ('syndrome'/exp OR syndrome)) OR (syndrome, AND sjogren) OR (sicca AND syndrome) OR (syndrome, AND sicca) OR (sjogrens AND syndrome)

#3 #1 AND #2

Table S2 Literature review of autoimmune diseases risk in endometriosis patients.

| **Objective** | **Results** | **Conclusion** | **Reference（PMID）** |
| --- | --- | --- | --- |
| To review the systematic reviews and Mendelian randomization studies that focused on the risk factors of systemic lupus erythematosus and shed light on the development of treatments for its prevention and intervention. | The results of systematic reviews showed that diseases (endometriosis, atopic dermatitis, allergic rhinitis), lifestyle (smoking, drinking, vaccination), and gene polymorphism influenced the incidence of systemic lupus erythematosus. | We should pay attention to preventing and treating systemic lupus erythematosus in patients with endometriosis, celiac disease, and periodontitis. | 37596678^[1]^ |
| To evaluate the association between endometriosis and SLE considering that both diseases may have an important and common immunological response, as well as relevant morbidity rates, healthcare costs, and prevalence. | A significant association between endometriosis and SLE was observed (RR = 2.47, 95% confidence interval: 1.33–4.59, P < 0.004, I2 = 54%). | The results suggest the existence of an association between endometriosis and SLE. | 35915351^[2]^ |
| To systematically review the literature on population-based studies investigating an association between endometriosis and autoimmune diseases and to conduct a meta-analysis of combinable results to investigate the extent and robustness of evidence. | Meta-analysis of a cross-sectional study, three case–control studies and two cohort studies has shown a significantly greater risk of SLE in women with endometriosis compared to control or comparison women (OR for cross-sectional study: 20.7 (95% CI: 14.3–29.9), P < 0.001; OR for case–control studies: 1.36 (95% CI: 1.07–1.73), P = 0.010, I2 = 49%; RR for cohort studies: 1.74 (95% CI: 1.10–2.77), P = 0.020, I2 = 0%). | The results suggest the existence of an association between endometriosis and SLE, RA and SS. | 31260048^[3]^ |
|  | Meta-analysis of two cross-sectional studies reported a significantly increased RA risk in women with endometriosis in comparison to the general female population (OR = 1.50 (95% CI: 1.18–1.91), P = 0.002, I2 = 0%). |  |  |
|  | In a cross-sectional study of US women, a greater risk of SS in women with endometriosis in comparison to the general US female population was reported (prevalence OR = 23.9 (95% CI: 15.5–36.5), P < 0.001). Meta-analysis of three case–control studies and a previously mentioned retrospective cohort study revealed a 76% greater odds of SS in association with endometriosis (OR for case–control studies: 1.76 (95% CI: 1.39–2.21), P < 0.001, I2 = 0%; SIR for cohort studies: 1.6 (95% CI: 1.3–2.0), P < 0.001) |  |  |
| To outline the roles of IL-17 in endometriosis, present the regulatory mechanism of IL-17 expression in endometriosis, identify the biological function (regulation of ectopic endometrial lesions, recruitment and function regulation of immune cells, and angiogenesis) of IL-17 in endometriosis, and discuss prospects in the potential treatment of these patients as well. | Commonly detected in PF and blood, the level of IL-17 rises in endometriosis patients. The regulation of IL-17 on ectopic endometrial involves multiple mechanisms, such as estrogen, cytokine, lnRNA and microRNA, HIF-1 and hypoxia, and might be more important in the initiation, but not in the later process of endometriosis. Moreover, IL-17 could be the stimuli that mediates the recruitment and activation of immune cells such as macrophages and neutrophils to facilitate the immune escape of ectopic endometrial cells, promote the progress of endometriosis, and contribute to the unexplained infertility . | The secretion of IL-17 increases under the regulation of ectopic microenvironment and other factors, and then IL-17 is deeply involved in endometriosis in the regulation of immune microenvironment, the invasion and growth of ectopic lesions. | 35582411^[4]^ |
| To compare the changes in inflammation and ANS in endometriosis, IBD, and RA; and to explore the role and possible mechanism of sympathetic and parasympathetic nerves in endometriosis-associated inflammation by referring to IBD and RA studies to provide some reference for further endometriosis research and treatment. | The level of PGE2 is increased in synovial fluid of RA patients and in PF of endometriosis women. Similar to endometriotic PF and lesions, the concentration of M2 macrophages is increased in synovial tissues. In addition, sympathetic nerve fibers are decreased and BDNF-positive cells are increased in RA synovium. | The changes in dysregulation of inflammatory factors as well as the function and innervation of the autonomic nervous system (ANS) have similar influence in endometriosis and some chronic inflammatory autoimmune diseases (AIDs) such as inflammatory bowel disease (IBD) and rheumatoid arthritis (RA). | 32145751^[5]^ |
| To review the effects of existing immunomodulators on the development of endometriosis, including immune cell regulators and immune factor regulators. | The onset and development of endometriosis are closely related to the abnormal function of the female autoimmune system, especially the function of some immune cells such as the aggregation of neutrophils, abnormal differentiation of macrophages, decreased cytotoxicity of NK cells, and abnormal function of T- and B-cell lines. Therefore, immunotherapy is probably a novel therapeutic strategy for endometriosis besides surgery and hormone therapy. | Abnormal immune response mediated by immune cells causes ectopic growth of endometrial cells and several immunologic factors are probably involved in the pathogenesis of endometriosis. | 37138868^[6]^ |
| To cast a look at endometriosis as a chronic and progressive gynecological disease. | Fresh endometriotic lesions are associated with induction of an inflammatory reaction represented by overproduction of prostaglandins (PGE2), metalloproteinases (MMP-2, -3, -9), cytokines (IL-1β, IL-8, IFN-γ, TNF-α, MCP-1 and MIF) and adhesive molecules (ICAM-1, VCAM-1) and activation of synthesis of reactive oxygen and nitrogen species. The inflammatory process may lead to defective folliculogenesis by an altered follicular milieu. An increase in the number and change in function of macrophages, T- and B-lymphocytes and reduction of NK cells have been reported. Treg lymphocytes are known to play an extremely important role in controlling and modulating changes in the aberrant immune response in endometriosis. Dysregulation of the immune system results in both increased progression of endometriosis and its severity. In inflammatory conditions the immune cells provide immune defense at the local level – in peritoneal fluid – and could further cause: 1) a decrease of the number of NK CD16+ cells with expression of KIRs and an increase of NK CD57+; 2) increased numbers of CD8+ cells and CD11b– immature dendritic cells; 3) an increase of FoxP3 expression in the regulatory T cell (Treg) population; 4) an increase of macrophages activating T- and B-lymphocytes leading to elevated synthesis of cytokines and/or autoantibodies. | Endometriosis resembles an immunodependent disease with the autoimmune background and breakdown of immunosuppressive mechanisms. | 35600152^[7]^ |
| To assess the current and past literature for efficacious non-invasive diagnostic markers for earlier detection of endometriosis. | / | Various anti endometrial antibodies may offer useful diagnostic tools. Anti-SLP2, anti-TMOD3, anti-TPM3, and anti-PDIK1L are particularly useful for early diagnosis in minimal to mild endometriosis. Anti-alpha enolase could also be used but yields results similar to CA125. Other non anti endometrial antibodies like anti-IMP1, anti-CA, aCL, anti-STX5 may be used as additional non-invasive diagnostic tools. Anti-TPO may be beneficial in patients in endometriosis patients with concurrent polycystic ovaries syndrome (PCOS). | 33722753^[8]^ |
| To review the current understanding between autoimmunity and EMS to provide important knowledge to develop future potential immunomodulatory therapy for the treatment of EMS. | / | The aberrant changes in cellular immune response and its cytokines are found to be related to the pathophysiology (immune escape, adhesion, invasion, angiogenesis and proliferation). Also, the presence of autoantibodies is another consequence of dysfunction of immune system. These immunological alterations cause decreased fecundity or even infertility by affecting endometrial receptivity, follicular fluid, sperm mobility and embryo cytotoxicity. Endometriosis and autoimmune disease share several similar characteristics, such as female (and hormonal) predominance, genetic polymorphisms, immunological abnormalities and chronic condition. | 30107265^[9]^ |
| summarizing the available epidemiological findings on the associations between endometriosis and other chronic diseases and discussing hypotheses for underlying mechanisms, potential sources of bias and methodological complexities. | We identified 9 studies on the links between endometriosis and autoimmune diseases. Endometriosis patients were reported to be at higher risk of some autoimmune diseases. | Increasing evidence suggests that endometriosis patients are at higher risk of several chronic diseases. Although the underlying mechanisms are not yet understood, the available data to date suggest that endometriosis is not harmless with respects to women's long-term health. | 25765863^[10]^ |
| To review the association between autoimmunity and endometriosis. | / | Endometriosis may coexist with autoimmune disease. The presence of autoreactive antibodies in the serum of some patients with endometriosis may be a natural by-product of inflammation and local tissue destruction. The finding of antibodies to ovarian and endometrial nuclear antigens in patients with endometriosis supports the concept of endometriosis being a multiple antibody autoimmune condition. These autoreactive antibodies may play a role in pregnancy loss and infertility in patients with endometriosis. Potential biomarkers such as some cytokines and autoantibodies, which are specifically upregulated in endometriosis, may be used to develop non-invasive diagnostic tools. | 22330229^[11]^ |
| To review the association between endometriosis and autoimmune disease and describes the potential role of inflammation in the development of immunologic self-reactivity in these patients | / | Symptomatic cases have been associated with progesterone resistance and dysregulated cytokine production in both ectopic and eutopic endometrium. This seems to be associated with chronic local inflammation and antibody self-reactivity. Coexistence of endometriosis with autoimmune disease has been documented in a small number of cases. The presence of autoreactive antibodies in the serum of some patients with endometriosis may be a natural byproduct of inflammation and local tissue destruction. | 20436316^[12]^ |
| To review the recent advances of the aetiology and pathogenesis of endometriosis and their implications for diagnostic and therapeutic innovations that will probably appear in clinical practice in the future. | / | Endometriosis remains an underdiagnosed disorder with substantial associated morbidity. Challenges for the future include non-invasive methods to diagnose the disorder, targeted drug discovery, and delineation of the genetics and genomics governing development of disease, contributions from the environment, and involvement of the immune system.The patient’s history, environmental exposures, family history, and physical examination continue to be important in the assessment and care of women with endometriosis. | 15541453^[13]^ |
| To discuss the multiple processes underlying the complex pathogenesis of endometriosis, with particular emphasis on the role played by the immune system with the induction of autoimmunity | / | There is now significant evidence that immune mediators such as cytokines, as well as NK cells and macrophages, might be involved in the patho-genesis of the disease. These data support the idea that endometriosis can be considered as an autoimmune disorder, associated with a dysfunction of natural immunity. | 12763528^[14]^ |
| To review the literature on the role ofautoimmunity in the etiology of endometriosis, compare the similarities in the pathophysiologies between endometriosis and autoimmune diseases, and discuss the use of immunomodulators currently used to treat autoimmune diseases as potential therapies for endometriosis. | Endometriosis shares many similarities with autoimmune diseases such as rheumatoid arthritis, Crohn’s disease, and psoriasis. These similarities include elevated levels of cytokines, decreased cell apoptosis, and T- and B-cell abnormalities. Because the use of immunomodulators and inflammatory modulators has proven to be an effective means of medical management for these autoimmune diseases, similar therapies may prove useful in treating endometriosis. | Although substantial evidence indicates that endometriosis at least shares many similarities with autoimmune diseases, endometriosis is primarily treated by using compounds that induce a hypoestro-genic environment. A review of the literature combined with the shortcomings of current means of medical management for endometriosis support the postulate that treatment of endometriosis with immunomodulators and inflammatory modulators is warranted. | 11476764^[15]^ |
| To describe endometrial and ovarian autoimmunity in women with endometriosis in 1981. | 1. The endometrial autoantigens to which the endometriosis patients have autoimmune reactions are endometrial transferrin and alpha 2-HS glycoprotein.  2. Levels of antibodies to both these proteins are specifically elevated in women with endometriosis, thus making them important candidates for developing an endometrial antibody assay for a non-invasive diagnosis of endometriosis.  3. Levels of transferrin and alpha 2-HS glycoprotein are significantly elevated in the peritoneal fluid of women with endometriosis.  4. Antibodies to transferrin and alpha 2-HS glycoprotein also inhibit in vitro sperm motility. | It is possible that endometrial autoantibodies playing an important role in the infertility often associated with endometriosis. | 10994636^[16]^ |
| To a briefly review innate and adaptive immunity and discuss whether defective immunity is fundamental to the pathophysiology of endometriosis, is an artifact of faulty study design of published reports, or is simply an epiphenomenon |  | Immunologic mechanisms have been proposed to explain some of the apparent differences resulting in the development and pathogenesis of endometriosis. The hypoth esis that endometriosis is caused by defective immunity involving al tered clearance of menstrual debris within the peritoneal cavity remains speculative at this time because insufficient data exist currently to allow differentiation between cause versus effect mechanisms of observed endometriosis-associated immunologic phenomena. Conversely, the hy pothesis that immunity may be involved in the maintenance and pro gression of disease is less speculative but as yet not substantiated defini tively. | 9163768^[17]^ |

Reference:

1. Xiao XY, Chen Q, Shi YZ, Li LW, Hua C, Zheng H. Risk factors of systemic lupus erythematosus: an overview of systematic reviews and Mendelian randomization studies. Adv Rheumatol. 2023 Aug 18;63(1):42.
2. Ferrari-Souza JP, Pedrotti MT, Moretto EE, Farenzena LP, Crippa LG, Cunha-Filho JS. Endometriosis and Systemic Lupus Erythematosus: Systematic Review and Meta-analysis. Reprod Sci. 2023 Apr;30(4):997-1005.
3. Shigesi N, Kvaskoff M, Kirtley S, Feng Q, Fang H, Knight JC, Missmer SA, Rahmioglu N, Zondervan KT, Becker CM. The association between endometriosis and autoimmune diseases: a systematic review and meta-analysis. Hum Reprod Update. 2019 Jul 1;25(4):486-503.
4. Shi JL, Zheng ZM, Chen M, Shen HH, Li MQ, Shao J. IL-17: an important pathogenic factor in endometriosis. Int J Med Sci. 2022 Apr 11;19(4):769-778.
5. Wei Y, Liang Y, Lin H, Dai Y, Yao S. Autonomic nervous system and inflammation interaction in endometriosis-associated pain. J Neuroinflammation. 2020 Mar 7;17(1):80.
6. Li W, Lin A, Qi L, Lv X, Yan S, Xue J, Mu N. Immunotherapy: A promising novel endometriosis therapy. Front Immunol. 2023 Apr 17;14:1128301.
7. Chopyak VV, Koval HD, Havrylyuk AM, Lishchuk-Yakymovych KA, Potomkina HA, Kurpisz MK. Immunopathogenesis of endometriosis - a novel look at an old problem. Cent Eur J Immunol. 2022;47(1):109-116.
8. Greenbaum H, Galper BL, Decter DH, Eisenberg VH. Endometriosis and autoimmunity: Can autoantibodies be used as a non-invasive early diagnostic tool? Autoimmun Rev. 2021 May;20(5):102795.
9. Zhang T, De Carolis C, Man GCW, Wang CC. The link between immunity, autoimmunity and endometriosis: a literature update. Autoimmun Rev. 2018 Oct;17(10):945-955.
10. Kvaskoff M, Mu F, Terry KL, Harris HR, Poole EM, Farland L, Missmer SA. Endometriosis: a high-risk population for major chronic diseases? Hum Reprod Update. 2015 Jul-Aug;21(4):500-16.
11. Eisenberg VH, Zolti M, Soriano D. Is there an association between autoimmunity and endometriosis? Autoimmun Rev. 2012 Sep;11(11):806-14.
12. Barrier BF. Immunology of endometriosis. Clin Obstet Gynecol. 2010 Jun;53(2):397-402.
13. Giudice LC, Kao LC. Endometriosis. Lancet. 2004 Nov 13-19;364(9447):1789-99.
14. Matarese G, De Placido G, Nikas Y, Alviggi C. Pathogenesis of endometriosis: natural immunity dysfunction or autoimmune disease? Trends Mol Med. 2003 May;9(5):223-8.
15. Nothnick WB. Treating endometriosis as an autoimmune disease. Fertil Steril. 2001 Aug;76(2):223-31.
16. Mathur SP. Autoimmunity in endometriosis: relevance to infertility. Am J Reprod Immunol. 2000 Aug;44(2):89-95.
17. Hill JA. Immunology and endometriosis. Fact, artifact, or epiphenomenon? Obstet Gynecol Clin North Am. 1997 Jun;24(2):291-306.
