# Supplementary material for: Causal effects of endometriosis on SLE, RA and SS risk: evidence from meta-analysis and Mendelian randomization
Source: BMC Pregnancy Childbirth. 2024 Feb 23;24:162. doi: 10.1186/s12884-024-06347-9 (PMC10885476; doi:10.1186/s12884-024-06347-9)
Supplement: Supplementary file 3 — Supplementary Material 3. [file 12884_2024_6347_MOESM3_ESM.pdf]

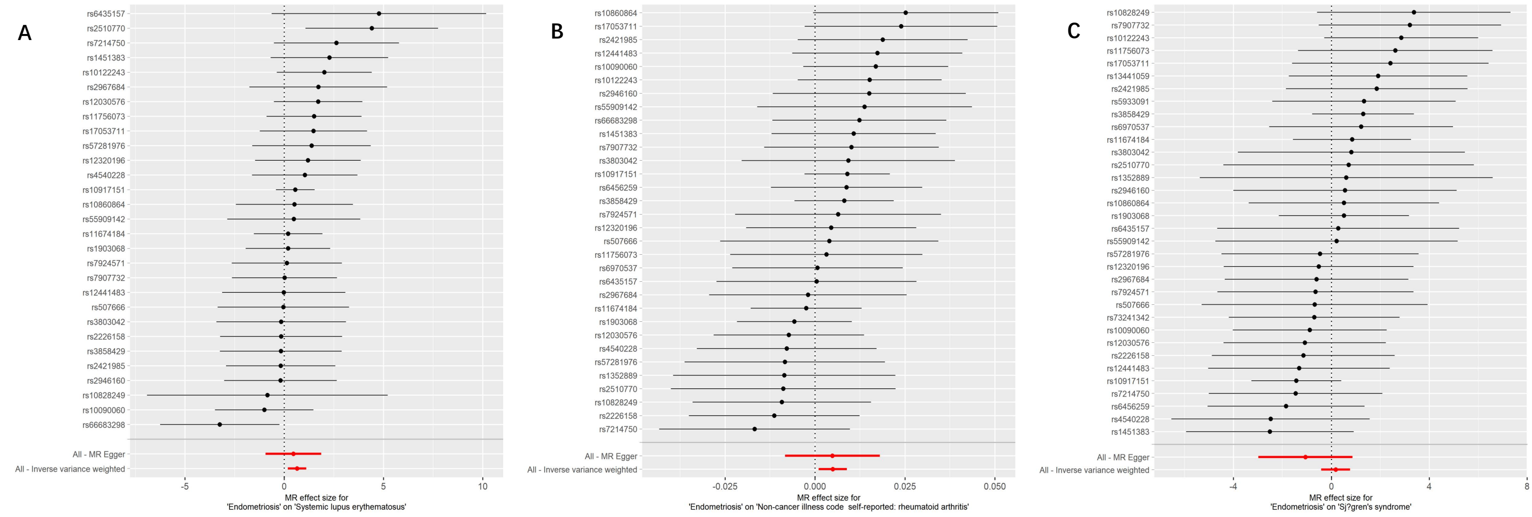

Figure S1 Forest plot for each SNP
(A)SLE; (B) RA; (C) SS.

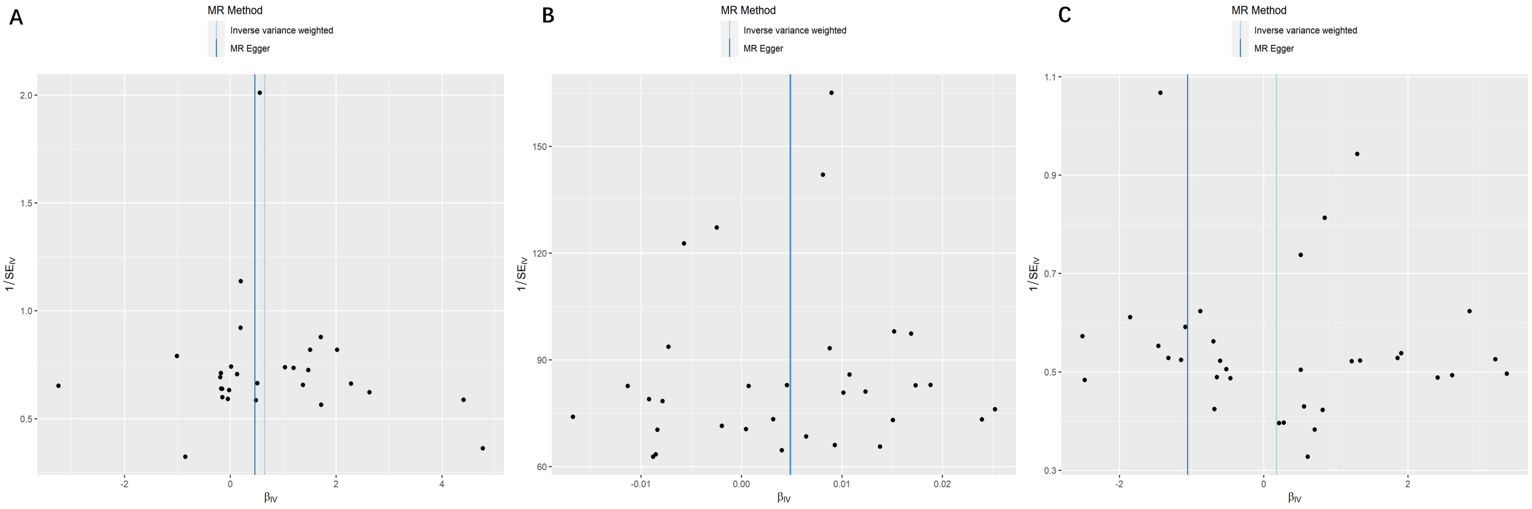

Figure S2 Funnel plots of the causal effect of endometriosis on autoimmune diseases
(A)SLE; (B) RA; (C) SS

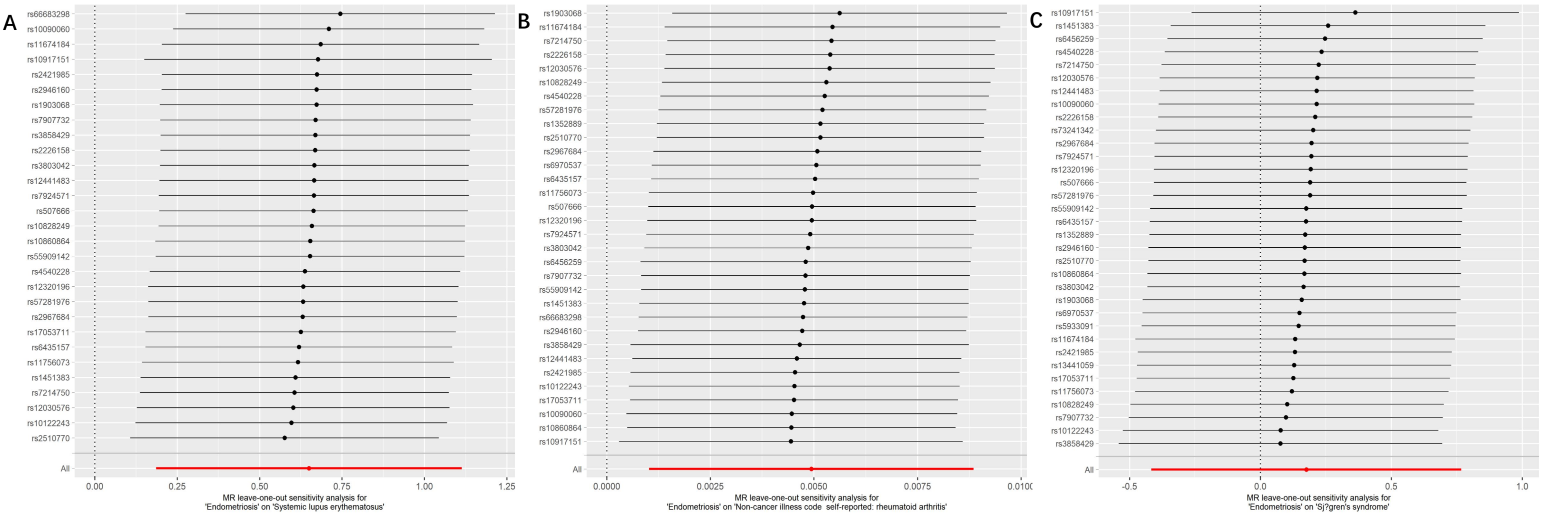

Figure S3 Leave-one-out analyses for endometriosis on autoimmune diseases
(A)SLE; (B) RA; (C) SS

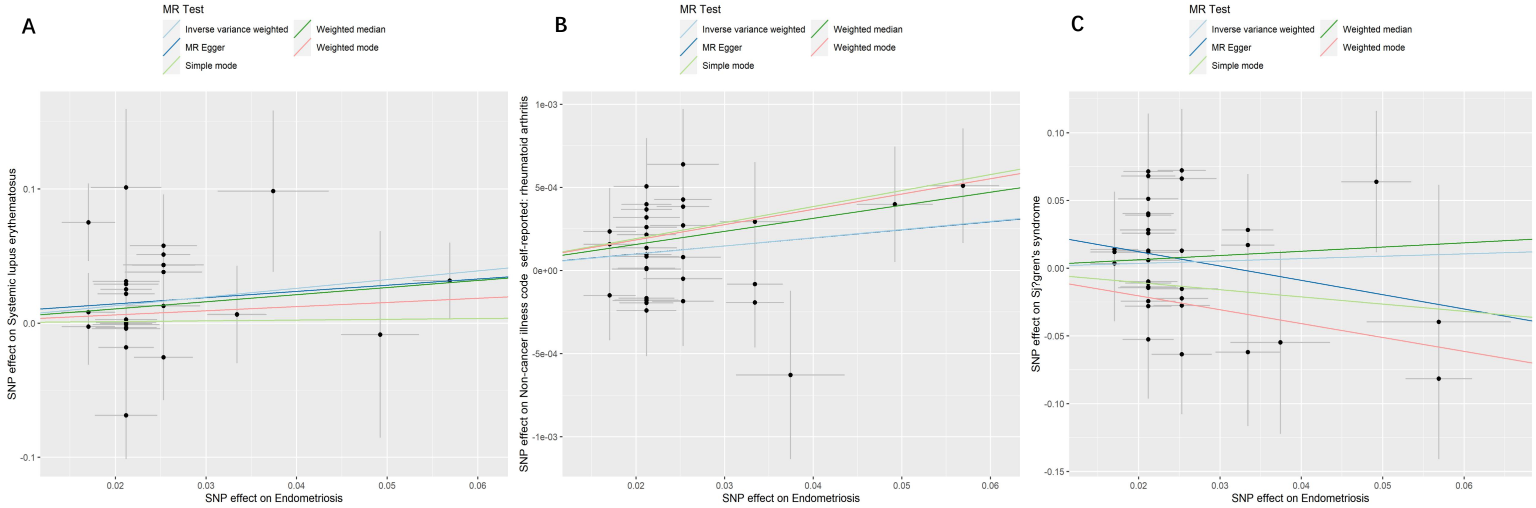

Figure S4 Scatter plots of the causal effect of endometriosis on autoimmune diseases
(A)SLE; (B) RA; (C) SS
